# Evaluation of the Efficacy of PARP Inhibitors in Metastatic Castration-Resistant Prostate Cancer: A Systematic Review and Meta-Analysis

**DOI:** 10.3389/fphar.2021.777663

**Published:** 2021-12-17

**Authors:** Kan Wu, Jiayu Liang, Yanxiang Shao, Sanchao Xiong, Shuyang Feng, Xiang Li

**Affiliations:** Department of Urology, Institute of Urology, West China Hospital, Sichuan University, Chengdu, China

**Keywords:** metastatic castration-resistant prostate cancer, PARP inhibitor, homologous recombination deficient, *BRCA* mutation, meta-analysis

## Abstract

**Background:** Poly(ADP-ribose) polymerase (PARP) inhibitors have breakthrough designations for metastatic castration-resistant prostate cancer (mCRPC). We performed a meta-analysis of current clinical trials to evaluate the efficacy of PARP inhibitors in mCRPC patients based on their genetic status.

**Methods:** On August 2020, PubMed, Scopus, Embase, Cochrane Central Register of Controlled Trials, and Web of Science were searched for phase II/III clinical studies on PARP inhibitors in mCRPC patients. Data were extracted independently by two investigators and analyzed using Review Manager software version 5.3. Primary endpoints included overall response rate (ORR) and progression-free survival (PFS).

**Results:** Nine clinical trials were identified and analyzed for the clinical benefit of PARP inhibitors in mCRPC patients (*n* = 1,219). Pooled analyses demonstrated that PARP inhibitors could provide a significant improvement of ORR and PFS in patients with homologous recombination deficiency (HRD) when compared with non-HRD patients. Within the HRD subgroup, *BRCA* mutation patients achieved significantly higher ORR [odds ratio (OR): 9.97, 95% confidence interval (CI): 6.08–16.35] and PFS rates at 12 months (OR: 3.23, 95% CI: 1.71–6.10) when compared with *BRCA* wild-type patients. Furthermore, patients harboring HRD without *BRCA* mutations have a higher objective response after PARP inhibitor treatment compared with non-HRD patients.

**Conclusion:** PARP inhibitor is an effective treatment option for mCRPC patients with mutations in genes related to the HR DNA repair pathway when compared with non-HRD patients. In addition to *BRCA* mutations, other HRD-related gene aberrations may also be used as novel biomarkers to predict the efficacy of PARP inhibitors.

## Introduction

Metastatic prostate cancer is an incurable disease and has a poor survival, with a 5-year survival rate of 29.8% (Siegel et al., 2019). Although a clinically significant response to androgen-deprivation therapy, most patients eventually develop lethal metastatic castration-resistant prostate cancer (mCRPC). Modern systemic treatments, including abiraterone, enzalutamide, docetaxel, and novel androgen receptor (AR)-signaling inhibitors, only provide a median survival of 2.8 years for mCRPC patients. Meanwhile, these approved agents show different efficacy profiles and only modestly improve survival (Francini et al., 2019). With the advancement in genomics analysis, novel genomic features or druggable targets have been identified, which may contribute to broadening new therapeutic scenario for mCRPC.

DNA damage repair pathways are meaningful therapeutic targets for diverse cancer types. These repair pathways involve single-strand break repair via base excision repair pathway, and double-strand break repair by nonhomologous end joining and homologous recombination (HR) pathways (Hoeijmakers, 2001). The poly(ADP-ribose) polymerase (PARP) is an important enzyme involved in the repair of DNA single-strand breaks. When the PARP function is inhibited, it will cause the accumulation of single-strand DNA breaks and subsequently lead to unrepaired DNA double-strand breaks (Dantzer et al., 2000). In normal cells, these breaks can be repaired through the HR repair pathway during cell cycle late S to G2 phase (McCabe et al., 2006). However, in cancer cells with tumor-specific HR deficiency (HRD), unrepaired double-strand DNA breaks after PARP inhibition treatment will persistently accumulate and eventually lead to tumor cell death. This phenomenon is called “synthetic lethality.” HRD mutations are results of the alterations in multiple gene pathways, including *BRCA1/2*, *ATM*, *ATR*, *CHK1/2*, *CDK12*, *FANCD2*, *PALB2*, and *RAD51/54* genes that might be associated with PARP inhibition sensitivity (Cancer Genome Atlas Resea, 2015; Pritchard et al., 2016). The development of CRPC is frequently accompanied by the accumulation of DNA damage repair gene mutations that lead to the survival and proliferation of CRPC cells (Grasso et al., 2012; Li et al., 2014). Around 12%–27% of metastatic prostate cancer patients carry deleterious mutations in the HR genes (Pritchard et al., 2016; Armenia et al., 2018). This provides a weakness for tumors, which can be exploited by PARP inhibition to induce selective cancer cell apoptosis.

There are currently several PARP inhibitors being tested in clinical trials for the treatment of mCRPC patients (Adashek et al., 2019). Based on the promising results observed in clinical trials, olaparib was approved by the US Food and Drug Administration (FDA) for the treatment of mCRPC patients with mutations in genes related to the HR DNA repair pathway. Thus, in this systematic review and meta-analysis, we aimed to investigate the clinical benefits of PARP inhibitors, administered alone or combined with AR signaling inhibitors, chemo- or immune therapies, in mCRPC patients compared with standard of care based on available clinical trial data. We also evaluated data on the efficacy of PARP inhibitor in HRD-positive tumors (including *BRCA* mutated or wild type with HRD) versus non-HRD tumors, to identify subgroups of patients who could benefit more from their use.

## Methods and Materials

### Search Strategy

In August 2020, a systematic literature search was conducted by screening the electronic databases (PubMed, Scopus, Embase, Cochrane Central Register of Controlled Trials, and Web of Science). The search strategies are as follows: {[(“Poly (ADP-ribose) Polymerase inhibitors” OR “PARP inhibitors”] OR “olaparib” OR “niraparib” OR “rucaparib” OR “veliparib” OR “talazoparib”} AND (“prostate”) AND (“randomized controlled trial” OR “clinical trial”). This meta-analysis was performed in line with the Preferred Reporting Items for Systematic Reviews and Meta-Analysis (PRISMA) guidelines without a time limit (Moher et al., 2010).

### Inclusion and Exclusion Criteria

Inclusion criteria: 1) The included studies must be phase II or Phase III clinical trials on PARP inhibitor as a single agent or in combination with other regimens in patients with mCRPC. 2) The primary endpoint of eligible studies was overall response rate (ORR) (≥50% PSA decline, or response according to Response Evaluation Criteria in Solid Tumors), or progression-free survival (PFS). 3) Only articles published in English were included.

Exclusion criteria: 1) Phase I clinical trial. 2) The excluded studies were case reports, retrospective studies, reviews or preclinical studies. 3) If the results of the same series are being continuously updated, we selected the latest publications. Finally, we excluded single-arm studies that did not report HRD status or only reported *BRCA* mutation or HRD carriers. The relevant articles were assessed based on eligibility criteria by two independent reviewers (K. Wu and J. Y. Liang) and disagreements were resolved by consensus or a third investigator (X. Li).

### Data Extraction

Two investigators (K. Wu and J.Y. Liang) independently extracted data of eligible studies. The following information was included: first author, publication year, study design, trial phase, ClinicalTrial.gov number, sample size, type of intervention/control, *BRCA* or HRD status, and primary endpoint (ORR or PFS).

### Risk of Bias Assessment

The quality assessment of eligible studies was independently assessed by two reviewers (K. Wu and J.Y. Liang) using the Cochrane Risk of bias tool, which included selection bias (sequence generation, allocation concealment), performance bias (blinding of participants and personnel), detection bias (blinding of outcome assessors), attrition bias (incomplete outcome data), reporting bias (selective outcome reporting), and other possible sources of bias. The risk level was graded as high, low, or unclear. Any disagreement was resolved by consensus or a third reviewer (X. Li).

### Statistical Analysis

The primary endpoint of this study was to evaluate the antitumor efficacy of PARP inhibitors in mCRPC patients, including ORR, 6- or 12-month PFS rate (PFS6 or PFS12). We also performed exploratory analysis; patients were regrouped into HRD carcinomas (*BRCA* mutated or wild type) and non-HRD carcinoma groups based on their BRCA mutation or HRD status.

The odds ratio (OR) with a 95% confidence interval (CI) was calculated to compare treatment effect. The comparison was considered statistically significant when *p* < 0.05. The statistical heterogeneity of results among studies was quantified by I^2^ statistic and chi-squared test. A random effects model was applied when heterogeneity was observed (I^2^ value >50% and *p* value <0.05); otherwise, a fixed effect model was adopted. The results of each study and pooled analyses were graphically displayed by forest plots. A two-tailed *p* value <0.05 was considered statistically significant. Funnel plot was visually generated to detect publication bias or small-study effect. All statistical analyses were performed by using Review Manager software version 5.3 (Cochrane).

## Results

### Study Selection and Characteristics

**FIGURE 1:**
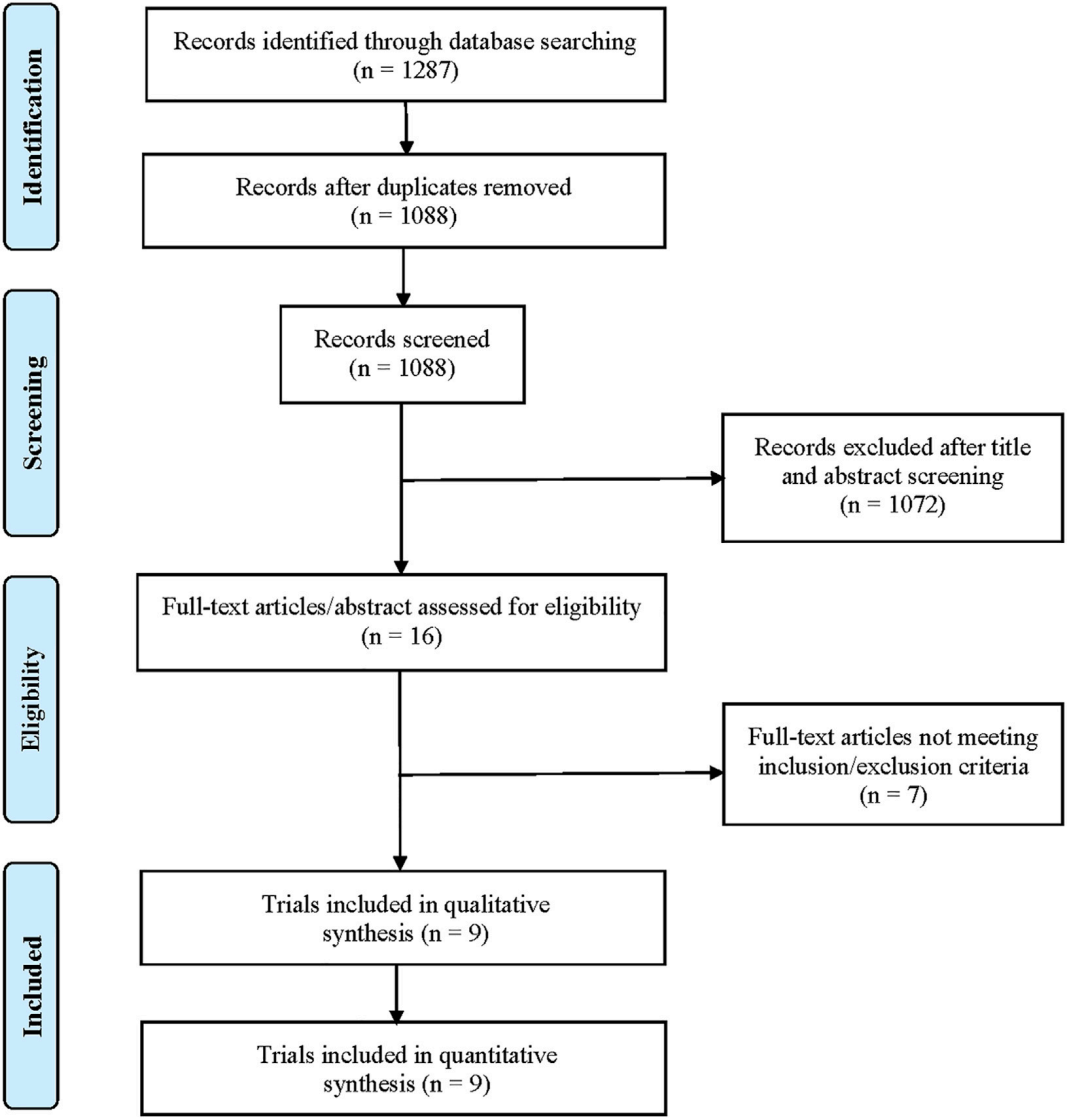
Flow diagram of study inclusion and exclusion.

A total of 1,287 potentially relevant records were retrieved through electronic search. After removing duplicates, a total of 1,088 unique records were identified for screening (Figure 1). After preliminary screening of titles and abstracts, only 16 clinical studies were further scrutinized for their eligibility. According to the inclusion/exclusion criteria, seven studies were succeedingly excluded for the following reasons: Three single-arm studies did not report HRD or *BRCA* mutation status, two studies reported results from the same cohort, one study was a clinical phase I trial, and one study was a retrospective study. Finally, nine clinical trials met the selection criteria, including three randomized controlled trials (Clarke et al., 2018; Hussain et al., 2018; de Bono et al., 2020) and six single-arm trials (Mateo et al., 2015; Karzai et al., 2018; Smith et al., 2019; Abida et al., 2020a; Abida et al., 2020b; Mateo et al., 2020; de Bono et al., 2021). Two of them (Mateo et al., 2015; Mateo et al., 2020) described the results from the TOPARP-B trial. Both studies were included in our analysis because the most recent publication (Mateo et al., 2020) focused on mCRPC patients with DNA repair gene aberrations and recruited more patients. However, we did not include both publications into the same pooled analysis to avoid statistical influences on research weights. In addition, two studies described the results of different populations in the TRITON2 trial (Abida et al., 2020a; Abida et al., 2020b). One reported the outcomes of patients with *BRCA* wild-type HRD (Abida et al., 2020a), and the other reported the outcomes of patients with a deleterious *BRCA* alteration (Abida et al., 2020b).

The characteristics of the included studies and enrolled patients are listed in Table 1. All clinical trials reported the clinical benefit of PARP inhibitors alone or in combination with AR signaling inhibitors or immune-checkpoint inhibitors in patients with mCRPC, ranging from 17 to 387 patients per study. Globally, a total of 1,219 patients were analyzed in this study and classified as the non-HRD (*n* = 139) and the HRD group (*n* = 926) based on the confirmed HRD status (deletions in genes involved in the HR DNA repair pathway). According to HRD and *BRCA* status, these patients with HRD-positive tumors were furthermore divided into the the *BRCA* mutant HRD subgroup (*n* = 418) and *BRCA* wild-type HRD subgroup (*n* = 508).

**TABLE 1:** Characteristics of the eligible studies.

Study (year)	Study name (NCT number)	Phase	Study design	Study drug	Total no. of patients	No. of HRD patients	No. of *BRCA*m patients	No. of *BRCA*wt patients	No. of non-HRD patients
Mateo et al. (2015)	TOPARP-B (NCT01682772)	II	Single arm	Olaparib	49	16	7	9	33
Clarke et al. (2018)	NCT01972217	II	RCT	Olaparib + abiraterone vs placebo + abiraterone	142	21	6	15	35
Hussain et al. (2018)	NCT01576172	II	RCT	Veliparib + abiraterone vs abiraterone	148	20	7	13	60
Karzai et al. (2018)	NCT02484404	II	Single arm	Olaparib + durvalumab	17	6	3	3	11
Abida et al. (2020a)	TRITON2 (NCT02952534)	II	Single arm	Rucaparib	193	193	115	78	0
Smith et al. (2019)	GALAHAD (NCT02854436)	II	Single arm	Niraparib	81	81	46	35	0
de Bono et al. (2020)	PROfound (NCT02987543)	III	RCT	Olaparib vs. abiraterone or enzalutamide	387	387	141	246	0
de Bono et al. (2020)	TALAPRO-1 (NCT03148795)	II	Single arm	Talazoparib	104	104	61	43	0
Mateo et al. (2020)	TOPARP-B (NCT01682772)	II	Single arm	Olaparib	98	98	32	66	0

Note. NCT, ClinicalTrials.gov identifier; *BRCA*m, *BRCA* mutation; *BRCA*wt, *BRCA* wild type; HRD, homologous recombination deficiency.

In the interventional arm, PARP inhibitors as monotherapy included: olaparib in three studies, rucaparib in one study, niraparib in one study, and talazoparib in one study. Combination therapies included olaparib plus abiraterone (one study), veliparib plus abiraterone (one study), and olaparib plus durvalumab (one study). All patients recruited in the clinical trials had been previously treated with standard chemotherapy or AR signaling inhibitors.

### Evaluation of the efficacy of Poly(ADP-ribose) Polymerase Inhibitor in Metastatic Castration-Resistant Prostate Cancer

#### PARP inhibitors vs. control

To evaluate the effectiveness of PARP inhibitors in mCRPC patients, we first conducted a meta-analysis to compare the clinical efficacy between the PARP inhibitor group and the control group. Three randomized controlled trials were eligible for the analysis of ORR or PFS, including 403 participants who received PARP inhibitor treatment and 274 participants receiving control therapies.

**FIGURE 2:**
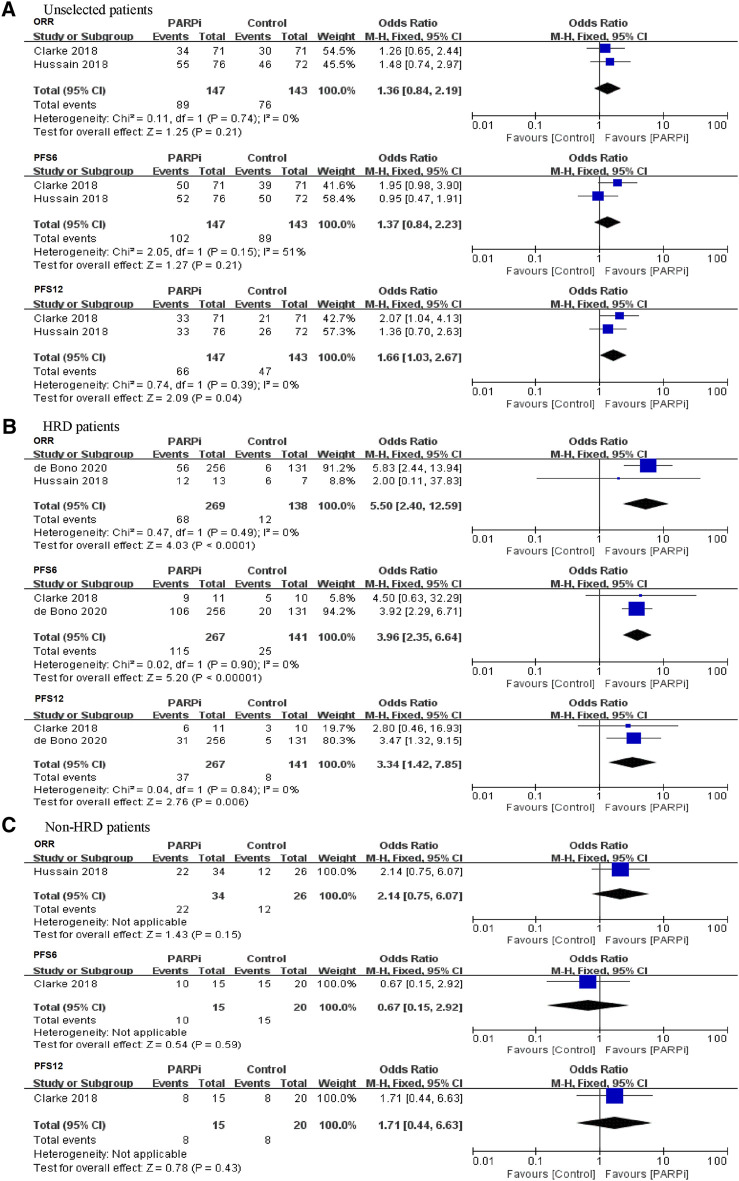
Forest plots of pooled analyses for poly(ADP-ribose) polymerase inhibitors (PARPi) vs. control treatments on overall response rate (ORR) and progression-free survival (PFS) in **(A)** unselected patients, **(B)** homologous recombination deficiency (HRD) patients, and **(C)** non-HRD patients.

For unselected patients irrespective of HRD status, there was no significant difference in the incidence of achieving ORR between the PARP inhibitor group and the control group (OR = 1.36, 95% CI: 0.84–2.19; Figure 2A). In terms of PFS, PARP inhibitors had no impact on PFS6 (OR = 1.37, 95% CI: 0.84–2.23) but had weaker improvements in PFS12 (OR = 1.66, 95% CI: 1.03–2.67) (Figure 2A).

In the HRD-positive group, the comparisons between PARP inhibitor group and control group showed that the PARP inhibitors significantly improved ORR (OR = 5.50, 95% CI: 2.40–12.59), PFS6 (OR = 3.96, 95% CI: 2.35–6.64), and PFS12 (OR = 3.34, 95% CI: 1.42–7.85) (Figure 2B). For the non-HRD group, only one study reported the treatment effect of PARP inhibitor vs. control on these patients. Although the results have less statistical power, it is interesting to observe no significant benefit in ORR and PFS from PARP inhibition in non-HRD patients compared with control therapy (Figure 2C).

#### Homologous Recombination Deficiency vs. non-Homologous Recombination Deficiency Metastatic Castration-Resistant Prostate Cancer

**FIGURE 3:**
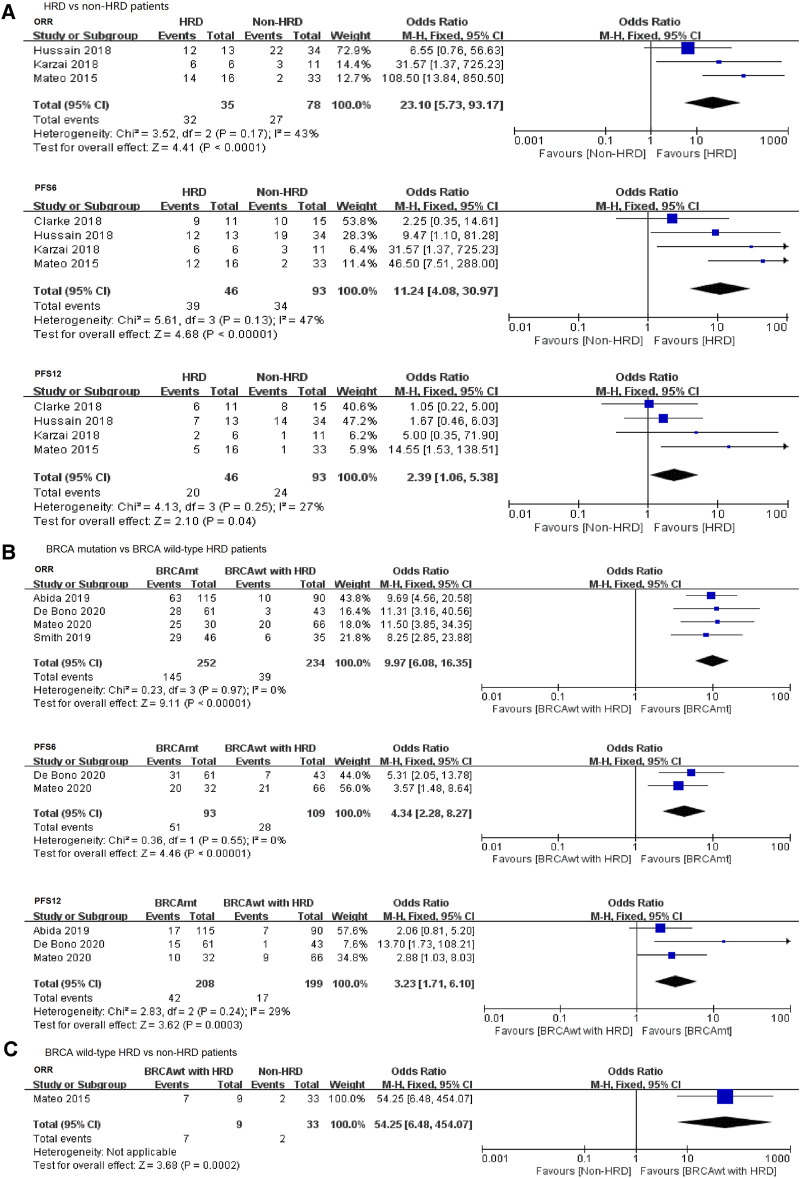
Forest plots of pooled analyses for the effect of PARP inhibitors on ORR and PFS in **(A)** HRD vs. non-HRD patients, **(B)** *BRCA* mutation vs. *BRCA* wild-type HRD patients, and **(C)** *BRCA* wild type with HRD vs. non-HRD patients.

To further compare the therapeutic effect of PARP inhibitions on the HRD and non-HRD populations, we subsequently performed a pooled analysis directly comparing these two groups based on the HRD status of the patients. Four studies were eligible and incorporated into an exploratory meta-analysis, including two RCTs and two single-arm studies. The results revealed that the PARP inhibitor was more effective in the HRD group compared with that in the non-HRD group in terms of ORR (OR = 23.10, 95% CI: 5.73–93.17), PFS6 (OR = 11.24, 95% CI: 4.08–30.97), and PFS12 (OR = 2.39, 95% CI: 1.06–5.38) (Figure 3A).

Thus, our findings showed that mCRPC patients with HRD positive (including *BRCA* mutation and *BRCA* wild type with HRD) are more likely to benefit from PARP inhibitor treatments when compared with patients with HRD-negative tumors.

#### *BRCA* Mutation vs. *BRCA* Wild Type With Homologous Recombination Deficiency vs. Non-Homologous Recombination Deficiency

It is still unclear whether other DNA repair gene pathways (such as *ATM*, *ATR*, *PALB2*, or *CHEK2*) also play an important role in the treatment of PARP inhibitors in mCRPC patients, except for *BRCA1/2* mutations. Therefore, we conducted a meta-analysis comparing patients with *BRCA* mutation and *BRCA* wild-type HRD positive. Four studies reported the ORR or PFS data of PARP inhibitors in both groups and could be pooled into this analysis. We observed that PARP inhibitors conferred a significant benefit on ORR and PFS in the *BRCA* mutation patients when compared with the *BRCA* wild-type HRD-positive patients, with an OR_ORR_ of 9.97 (95% CI: 6.08–16.35), OR_PFS6_ of 4.34 (95% CI: 2.28–8.27), and OR_PFS12_ of 3.23 (95% CI: 1.71–6.10) (Figure 3B).

With regard to the analysis of the *BRCA* wild-type HRD-positive group vs. the non-HRD group, only one article mentioned ORR in the two groups, and the statistical power is low. Strikingly, we observed a significant difference in ORR when comparing the *BRCA* wild-type HRD-positive patients with the HRD-negative patients (Figure 3C), suggesting a potential benefit from PARP inhibition treatment in other HRD gene aberration subgroup.

### Exploratory Analyses

**FIGURE 4:**
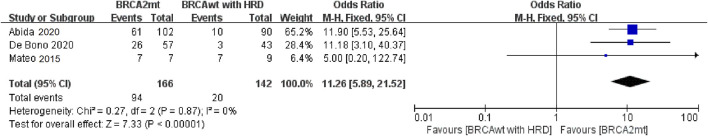
Forest plots of pooled analyses for the effect of PARP inhibitors on ORR in *BRCA2* mutation vs. *BRCA* wild-type HRD patients; Abbreviations: HRD, homologous recombination deficiency; ORR, overall response rate; PFS6/12, progression-free survival rates at 6/12 months.

Because *BRCA1* is a relatively rare mutation in mCRPC (*BRCA1*, 2%; *BRCA2*, 10%), and the data from PROfound trial showed that PARP inhibitors have very different efficacy in *BRCA1* and *BRCA2* mutations in mCRPC, in this study, we tried to separate *BRCA1* and *BRCA2* in the analysis. Three studies were pooled into this analysis comparing patients with *BRCA2* mutation and *BRCA* wild-type HRD. Subgroup analysis demonstrated that PARP inhibitors could provide a significant improvement in ORR to *BRCA2* mutated patients in comparison with *BRCA* wild-type HRD patients (OR = 11.26, 95% CI: 5.89–21.52) (Figure 4). Due to the lack of sufficient data and the small number of patients with *BRCA1* mutation, we did not perform a meta-analysis to evaluate the efficacy of PARP inhibitors in this population.

### Quality of Included Studies

The “risk of bias graph” showed that there was moderate selection bias in this meta-analysis because six of the nine clinical trials were single-arm studies (Supplementary Figure S1). The funnel plots for each pooled analysis suggested that there was a low risk of publication bias, even if the number of clinical studies is relatively low.

## Discussion

The results of this meta-analysis showed that PARP inhibitors could confer a significant improvement in tumor response and disease control survival for mCRPC patients with HRD carriers in terms of ORR and PFS. However, patients with non-HRD tumors did not derive a statistically significant benefit from PARP inhibitors compared with novel AR-targeted therapy. As growing evidence shows that mCRPC patients with other mutations in genes related to the HR DNA repair pathway (besides *BRCA1/2* mutations) also appear to benefit from PARP inhibitors (Mateo et al., 2015; Mateo et al., 2020), we further carried out a subgroup analysis and divided HRD patient population into the *BRCA* mutant group and *BRCA* wild-type group. Our results showed that the magnitude of benefits from PARP inhibitors varies greatly between these two subgroups. Compared with patients who harbored non-*BRCA* mutant HRD, a more vigorous efficacy on ORR and PFS upon PARP inhibitors was observed in *BRCA* mutation patients. Furthermore, we separated *BRCA1* and *BRCA2* in the subgroup analysis, and found that *BRCA2* is likely the most important mutation in prostate cancer, and the impact of BRCA1 needs to be clarified in future studies. Interestingly, we also observed a significant benefit in terms of ORR in *BRCA* wild-type HRD positive patients compared with non-HRD patients. These findings support the views that besides *BRCA* mutations, other non-*BRCA* HRD-related gene aberrations may also be used to predict the antitumor activity of PARP inhibitors.

Preclinical data and clinical practice results showed that *BRCA*-mutated cancers were more sensitive to PARP inhibitors than *BRCA* wild-type tumors, suggesting that this could be used as an excellent marker for selecting optimal candidates for PARP inhibitor therapy (Bryant et al., 2005; Farmer et al., 2005). However, in the TOPARP-A study by Mateo (Mateo et al., 2015), 33% (16/49) patients with unselected mCRPC had a confirmed response to PARP inhibitors, indicating that other key DNA repair genes might be functionally correlated with the sensitivity of PARP inhibitors. In the FDA breakthrough designation, an alteration in *ATM* gene was also included as a predictive biomarker for PARP inhibitor response. In addition, *BRCA* mutations account for only a small proportion of CRPC patients (Taylor et al., 2010; Grasso et al., 2012; Mateo et al., 2015; Robinson et al., 2015). Hence, only using *BRCA* mutational status as a marker for PARP inhibition sensitivity is insufficient, and it may miss a potentially larger proportion of responding patients. Following large-scale cancer sequence analysis, many other HRD-related genes (*CDK12*, *ATM*, *PALB2*) were commonly found in mCRPC (Cancer Genome Atlas Resea, 2015; Robinson et al., 2015; Pritchard et al., 2016), and these non-*BRCA* DNA repair genes could be used as alternative biomarkers to predict the sensitivity of PARP inhibitors. In this study, we also observed a significant improvement in tumor control of *BRCA* wild-type HRD patients compared with non-HRD patients, which may expand the benefit of PARP inhibitor therapy to more mCRPC patients.

Our study showed that patients with HRD-positive mCRPC could be more likely to benefit from PARP inhibitor treatment. However, the management of non-HRD mCRPC patients remains challenging without a novel effective therapy. Recent reports from preclinical models suggested that the synergistic effect of PARP inhibitors and other targeted drugs might provide an additional clinical benefit to patients with unselected mCRPC, irrespective of HRD status (Li et al., 2017; Kaplan et al., 2019). For example, the xenograft study by Likun et al. showed that AR inhibitor enzalutamide could suppress the expression of a specific set of HR genes (including *BRCA1*, *RAD51AP1*, *RAD51C*, *RAD54L*, and *RMI2*), thus, leading to the induction of HRD in CRPC cells. More importantly, they also found that this pharmaceutically induced HRD could synergize with the effects of PARP inhibitors to promote DNA damage-induced cell death and suppress xenograft tumor growth in mice (Li et al., 2017). As for PARP inhibitor combined immunotherapy, a recent study found that PARP inhibitor could increase the expression of programmed death ligand 1 (PD-L1) in DDR-mutated cells and facilitate the release of neoantigens due to DNA damage in tumors (Chabanon et al., 2019). The phase-Ib/II KEYNOTE-365 study (NCT02861573) showed promising clinical efficacy with pembrolizumab and olaparib in patients with unselected mCRPC (Yu et al., 2019). In addition, the vascular endothelial growth factor receptor tyrosine kinase inhibitor cediranib could suppress the expression of HRD-related genes (*BRCA1*, *BRCA2*, and *RAD51*) and enhance the antitumor activity of PARP inhibitors *in vitro* (Kaplan et al., 2019). A randomized phase 2 trial by Kim et al. confirmed that cediranib/olaparib combination therapy significantly improved radiographic PFS in unselected, mCRPC patients (Kim et al., 2020). In summary, the above results support more randomized clinical trials for the combination of PARP inhibitors and other drugs in unselected patients with mCRPC, which may expand the future clinical use of PARP inhibitors even in the absence of HRD.

There are several limitations in our meta-analysis. First, our study largely encompassed single-arm studies because of the limited number of randomized clinical trials currently available. Additionally, a comparison of the efficacy of PARP inhibitors in the *BRCA* wild type with HRD patients and non-HRD patients is less commonly studied, and this may make it impossible to fully assess the additional benefit of PARP inhibitors in patients with *BRCA* wild-type HRD. Finally, given the relatively small sample size in this study, randomized controlled trials with larger and longer clinical follow-up time are warranted, to evaluate the effect of PARP inhibitors and combination therapies on the long-term survival of mCRPC patients according to differential HRD genes.

## Conclusion

Our findings confirmed that mCRPC patients with mutations in genes related to the HR DNA repair pathway are more likely to benefit from PARP inhibitor treatment when compared with non-HRD patients, suggesting that HRD-related gene aberrations can be used as a predictive biomarker to guide clinical decision making. Also, based on the magnitude of benefit of PARP inhibitors and the genetic status of patients, we could rank the subgroups of mCRPC patients in the following order: *BRCA-*mutant HRD > HRD without *BRCA* mutation > non-HRD; these results can help identify a suitable subpopulation who may benefit from PARP inhibitors and determine an appropriate control arm for future clinical trials. In addition, more emphasis needs to be placed on the different roles of *BRCA1* and *BRCA2* mutations.

## Data Availability

The raw data supporting the conclusion of this article will be made available by the authors, without undue reservation.

## Supplementary Material

Supplementary Figure S1 Risk of bias graph.

## Abbreviations

HRD, homologous recombination deficiency; ORR, overall response rate; PARPi, PARP inhibitor; PFS6/12, progression-free survival rates at 6/12 months.
