# Supplementary figures and images for: Evaluation of the Efficacy of PARP Inhibitors in Metastatic Castration-Resistant Prostate Cancer: A Systematic Review and Meta-Analysis
Source: Front Pharmacol. 2021 Dec 17;12:777663. doi: 10.3389/fphar.2021.777663 (PMC8718674; doi:10.3389/fphar.2021.777663)

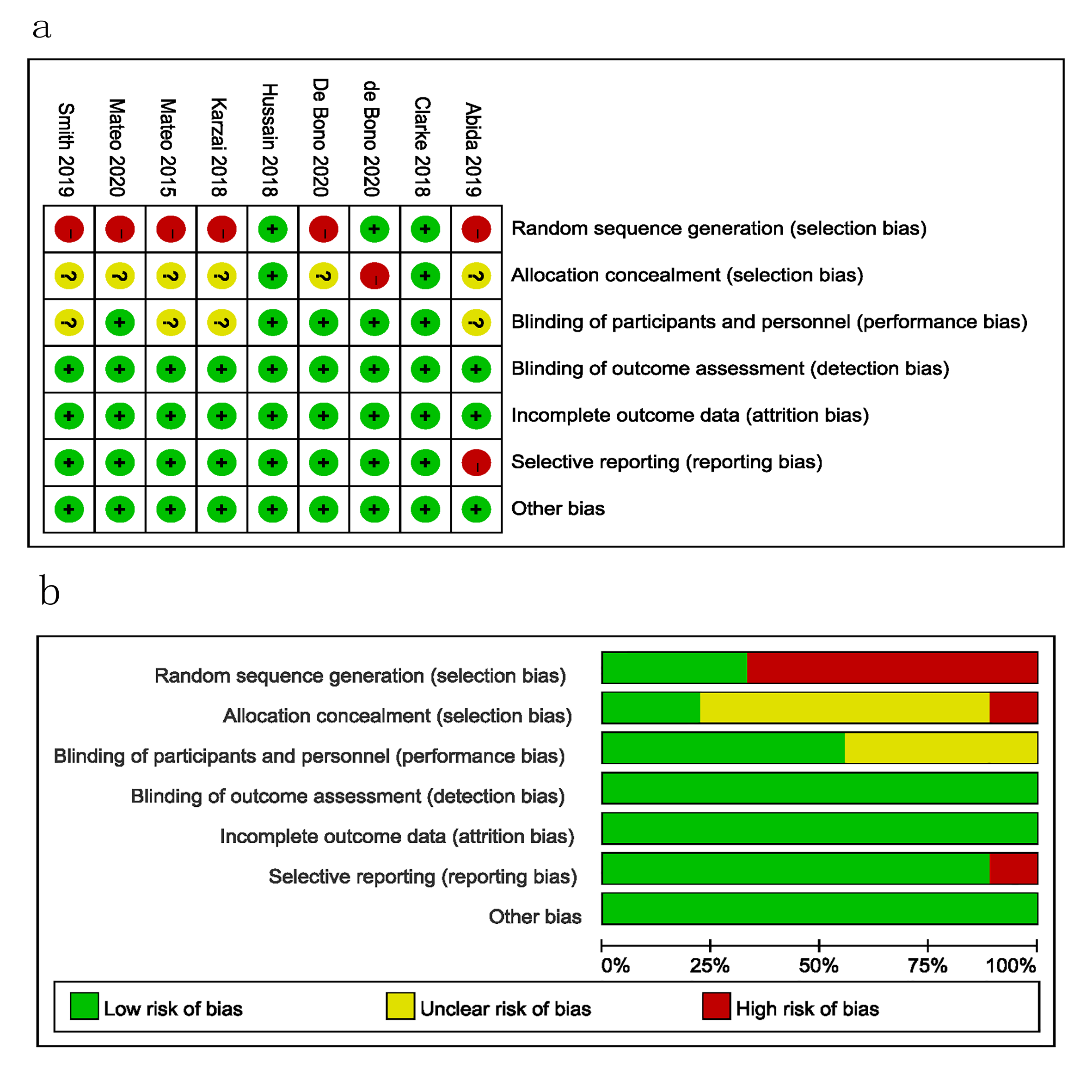

Supplement: Supplementary file 1 [file Image1.TIF]
